# Public Health–Driven Research and Innovation for Next-Generation Influenza Vaccines, European Union

**DOI:** 10.3201/eid2502.180359

**Published:** 2019-02

**Authors:** Adoración Navarro-Torné, Finnian Hanrahan, Barbara Kerstiëns, Pilar Aguar, Line Matthiessen

**Affiliations:** European Commission Directorate-General for Research and Innovation, Brussels, Belgium

**Keywords:** influenza, influenza pandemic, influenza vaccines, biomedical research, innovation, financial support, European Union, global health, public health, drug legislation, vaccines, viruses

## Abstract

Influenza virus infections are a major public health threat. Vaccination is available, but unpredictable antigenic changes in circulating strains require annual modification of seasonal influenza vaccines. Vaccine effectiveness has proven limited, particularly in certain groups, such as the elderly. Moreover, preparedness for upcoming pandemics is challenging because we can predict neither the strain that will cause the next pandemic nor the severity of the pandemic. The European Union fosters research and innovation to develop novel vaccines that evoke broadly protective and long-lasting immune responses against both seasonal and pandemic influenza, underpinned by a political commitment to global public health.

Influenza virus infection causes a highly contagious respiratory illness. Symptoms may be mild to severe, frequently leading to hospitalization and death. Population groups such as elderly persons, young children, pregnant women, and persons with certain chronic conditions are at particularly high risk, but anyone can suffer from serious complications. Each year seasonal influenza affects ≈10%–30% of the population of Europe (*1*). Latest studies estimate that, worldwide, 290,000–645,000 deaths result from seasonal influenza each year (*2*).

Influenza also poses a significant economic burden. In Europe, a macroeconomic model of an influenza pandemic in the EU-25 (the European Union [EU] minus Bulgaria, Croatia, and Romania) showed a fall in gross domestic product (GDP) of 1.6%. Long-term GDP would also fall by 0.6% due to a decreased labor force caused by the pandemic (*3*). In the United States the annual economic burden of influenza was estimated at US $5.79 billion in 2015 (*4*). US economic loss due to pandemic influenza has been estimated at up to US $45.3 billion in GDP losses (*5*).

Together, the human and economic cost of seasonal influenza alone is very great, and if another pandemic occurs these costs will escalate to untenable levels. For a vaccine-preventable disease this is an unusually high cost; in the United States, the economic burden of influenza accounts for 65% of the total estimated economic burden of all vaccine-preventable diseases (*4*).

Influenza viruses are members of the *Orthomyxoviridae* family and have a segmented negative-sense single-stranded RNA genome. Three genera of influenza viruses (A, B, and C) have infective capacity in humans, although A and B are the most common circulating strains. Influenza C infection is less frequent and usually produces mild disease, thus not constituting a relevant public health problem. Influenza viruses are classified according to antigenic differences in the viral nucleoprotein (NP) and matrix protein (M). Influenza type A viruses are further classified into subtypes by the combinations of 2 different proteins, hemagglutinin (HA) and neuraminidase (NA), anchored on the surface of the virus. The subtypes of influenza A viruses currently circulating among humans as seasonal influenza are influenza A(H1N1) (Ref) and A(H3N2) (*6*). Influenza B viruses can be sorted into 2 main groups or lineages, B/Yamagata and B/Victoria. Influenza viruses have high rates of evolution, and genetic mutations (genetic drift) or reassortments (genetic shift) can result in emerging influenza viruses with the potential to cause pandemics. Influenza A is also widespread in animals, such as birds, horses, dogs, and pigs. Several of the zoonotic strains of influenza A(H7N9) and A(H5N1) can also infect humans, but these strains are not currently endemic in humans. The species diversity of influenza provides the virus with numerous opportunities for reassortment between subtypes, and natural reservoirs of influenza A make elimination of the disease impossible.

Vaccination is the cornerstone for disease prevention, and influenza vaccines have existed since Jonas Salk and Thomas Francis made their breakthrough in 1938 (*7*). However, influenza viruses are continuously changing, resulting in antigenic shifts and drifts. For this reason, the characteristics of influenza viruses are closely monitored by the World Health Organization (WHO) Global Influenza Surveillance and Response System (GISRS), whereby countries’ national influenza centers biannually share representative viruses with WHO Collaborating Centres for reference and research on influenza (*8*).

Currently, licensed influenza vaccines are designed to produce antibodies against the viral HA protein. These strain-specific HA antibodies bind to the virus to prevent infection. There are 3 classes of licensed seasonal influenza vaccines. The first class is inactivated influenza vaccine (IIV), which can be either trivalent or quadrivalent. Trivalent vaccines contain H1N1 and H3N2 subtypes of influenza A, together with the predicted dominant lineage of influenza B for that season. Quadrivalent vaccines include subtypes H1N1 and H3N2, along with both influenza B lineages. The second class is the live attenuated influenza vaccine (LAIV), which contains the same 4 influenza strains as quadrivalent vaccines but is delivered in the form of an intranasal spray. The LAIV elicits a strain-specific serum IgG, as do IIVs, and also mucosal IgA and T cell responses. The third licensed vaccine class is a recombinant HA vaccine. This vaccine is egg-free, and its rapid manufacturing process makes it very useful at short notice, such as in the case of a pandemic (*9*). Indeed, pandemic preparedness requires a series of measures from funders, developers, and regulators to speed up the potential launch of a new vaccine. Several potential pandemic vaccines for influenza A(H5N1) have been licensed, for instance (*10*). These vaccines contain a strain of influenza that very few persons have been exposed to but that could potentially cause a pandemic. In this case, initiating a preliminary registration dossier can greatly shorten regulatory approval time if a pandemic does occur.

Despite existing for 80 years, influenza vaccines have substantial shortcomings related to their availability and effectiveness (*11*). The reasons include their production from embryonated eggs (*12*) and a lengthy manufacturing process. Another challenge is in the efficacy of the vaccines themselves, which is related to the immune response they elicit, in particular the waning of vaccine-specific antibodies, as well as the antigenic drift and the unpredictability of annual vaccine strain selection and the lack of accurate correlates of protection for influenza vaccines. The fact that current seasonal influenza vaccines fail to protect against drifted seasonal influenza viruses or novel pandemic viruses is a major issue. During recent influenza seasons, overall vaccine effectiveness has been as low as 19% (in 2014–15 in the Northern Hemisphere) (*13**,**14*). Moreover, effectiveness of influenza vaccines is particularly low in the elderly, a group that is most susceptible to the disease and its complications. Aging is associated with the progressive deterioration of the immune system, referred to as immunosenescence, and with a chronic low-grade proinflammatory state, inflammaging. These 2 interrelated phenomena are an important cause of low influenza vaccine effectiveness in this age group (*15**–**17*). In addition, uptake of seasonal influenza vaccine in risk groups in the WHO European Region is low and even declining in several EU countries (*18*).

Although the idea seems counterintuitive, recent studies point to a so-called pandemic paradox (*19*), finding that previous exposure to influenza virus strains can enhance susceptibility during pandemics. The occurrence of vaccine-associated antibody-dependent enhancement of viral infection is of concern (*20*) and has implications for the design of multiple boost vaccination schedules from a clinical perspective.

Low- and middle-income countries (LMICs) also face particular challenges with influenza. These countries often have restricted funding for vaccine provision and low or absent regional vaccine manufacturing capacity, which means they often have limited capacity to stockpile influenza vaccines or to respond adequately to seasonal or pandemic influenza (*21*). Surveillance data on the influenza burden in LMICs are limited, but a recent report suggested that seasonal influenza was associated with 28,000–111,500 deaths globally from acute lower respiratory infections in children <5 years of age and that 99% of these deaths occurred in LMICs (*22*). This stark imbalance highlights the need to improve influenza vaccination in a way that will benefit those who suffer the greatest burden. To address this situation, WHO, within the Pandemic Influenza Preparedness Framework, has reached agreements with vaccine manufacturers for pledges of over 400 million doses of pandemic vaccine in real time during the next pandemic (*23*).

## Paving the Way for Novel Influenza Vaccines

Ideally, new influenza vaccines are expected to provide long-lasting, broadly protective humoral and cellular immunity, aiming also to protect from pandemic influenza. The implications of these expectations are multifaceted and require great technological and innovative advancements (*24**,**25*).

The EU has fostered initiatives for novel influenza vaccine development through the seventh Framework Programme for Research and Technological Development (FP7) (Table 1) and, more recently in Horizon 2020, the EU’s current research and innovation program. FP7 funded 25 influenza vaccine–focused projects (Figure) that accounted for around €87 million, and an additional €18 million have been granted under Horizon 2020.

**Table 1:** Influenza vaccine–focused projects funded through the seventh Framework Programme for Research and Technological Development of the European Union*

Project acronym	Project title	Funding scheme	Research focus	Website	Total EU contribution, €
FP7 projects					
FLUCOP	Standardization and development of assays for assessment of influenza vaccine correlates of protection	Public private partnership	Immunoassays for correlates of protection	http://www.flucop.eu/	6,100,000
IMECS	Identification of mechanisms correlating with susceptibility for avian influenza	Translational collaborative research	Correlates of protection	http://cordis.europa.eu/project/rcn/88172_en.html	2,797,287
NASPANVAC	Nasal pandemic influenza vaccine	Translational collaborative research	Pandemic influenza vaccine	http://cordis.europa.eu/project/rcn/86777_en.html	3,573,648
INIMIN	Defense mechanisms of innate immunity against influenza virus	Training and mobility	Innate immunity to influenza	http://cordis.europa.eu/project/rcn/88512_en.html	225,715
PLAPROVA	Plant production of vaccines	Translational collaborative research	VLP-based influenza vaccine	http://cordis.europa.eu/project/rcn/89887_en.html	1,998,354
FLUPLAN	Novel strategies to combat future influenza pandemics	Frontier research	MVA-based influenza vaccine	http://cordis.europa.eu/project/rcn/94581_en.html	2,187,758
IMMUNExplore	New approaches to analyze and exploit the human B and T cell response against viruses	Frontier research	Innate immunity to influenza	http://cordis.europa.eu/project/rcn/95521_en.html	1,979,200
REPLIXCEL	Highly efficient new generation synthetic RNA replicon–based vaccine	Training and mobility	RNA replicon-based influenza vaccine	http://cordis.europa.eu/project/rcn/94667_en.html	2,591,172
FERFLU EXPRESS	Investigation of influenza immune responses and vaccine efficacy correlates by global expression profiling and immunological analyses in the ferret model of influenza	Training and mobility	Immune response to influenza vaccine	http://cordis.europa.eu/project/rcn/95964_en.html	239,693
SIMULATA	System for immunological modeling as an ultimate tool to link adjuvant function to adaptive immune responses	Training and mobility	Immune response to influenza vaccine	http://cordis.europa.eu/project/rcn/95337_en.html	220,500
FLUPIG	Pathogenesis and transmission of influenza virus in pigs	Translational collaborative research	Pandemic influenza vaccine	http://www.flupig.ugent.be/	4,854,452
TCDIFLU	Impact of adjuvants on T cell differentiation after influenza vaccination analyzed at the single cell level	Training and mobility	Immune response to influenza vaccine	http://cordis.europa.eu/project/rcn/98379_en.html	180,084
INFLUENZA PREDICTION	Phylogenetic prediction of the evolutionary trajectory of the influenza virus	Training and mobility	Seasonal influenza modeling	http://cordis.europa.eu/project/rcn/104037_en.html	209,033
ARCAS	ARCAS: Analysis of the route to commercialization of MVA-based influenza vaccines	Frontier research	MVA-based influenza vaccine	http://cordis.europa.eu/project/rcn/105989_en.html	149,840
MAJARVISCIG	A single dose, cytomegalovirus-based vaccine to induce heterosubtypic protective immunity to influenza A virus	Training and mobility	Immune response to influenza vaccine	http://cordis.europa.eu/project/rcn/107995_en.html	100,000
UNIVAX	A “universal” influenza vaccine through synthetic, dendritic cell–targeted, self-replicating RNA vaccines	Translational collaborative research	RNA replicon-based influenza vaccine	http://www.univax-fp7.eu/index.php?id = 1293	5,999,457
UNISEC	Universal influenza vaccines secured	Translational collaborative research	Influenza vaccine development platform	http://www.unisecconsortium.eu/	5,972,181
FLUTCORE	Development of a universal influenza vaccine based on tandem core technology	Translational collaborative research	Tandem core technology for influenza vaccine	http://www.flutcore.eu/	3,162,304
FLUNIVAC	Influenza virus universal vaccine development program	Translational collaborative research	MVA-based influenza vaccine	http://flunivac.eu/	5,066,881
EDUFLUVAC	Combinatorial immunization strategy to educate the immune system toward cross-recognition and coverage against antigenic drift in seasonal influenza virus exposure	Translational collaborative research	VLP-based influenza vaccine	http://www.edufluvac.eu/	4,647,149
UNIVACFLU	Universal flu vaccine	Training and mobility	European training platform for influenza vaccines	http://cordis.europa.eu/project/rcn/109828_en.html	4,116,701
IRINVAC	Immune responses to influenza vaccine	Training and mobility	Immune response to influenza vaccine	http://cordis.europa.eu/project/rcn/109387_en.html	100,000
FLU-MAL VLP	Chimeric influenza-VLP used as vaccine platform for presentation of foreign antigens: application to malaria antigens	Training and mobility	VLP-based influenza vaccine	http://cordis.europa.eu/project/rcn/186122_en.html	194,047
SAMUFLU	Self-amplifying RNA technology applied to the development of a universal influenza vaccine	Training and mobility	RNA replicon-based influenza vaccine	http://cordis.europa.eu/project/rcn/188000_en.html	187,415
ADITEC	Advanced immunization technologies	Translational collaborative research	Immune response to influenza vaccine	http://www.aditecproject.eu/	29,980,670
Horizon 2020 projects					
BROADimmune	Structural, genetic, and functional analyses of broadly neutralizing antibodies against human pathogens	Frontier research	Neutralizing antibodies	https://cordis.europa.eu/project/rcn/198722_en.html#result-217092	1,867,500
DRIVE	Development of robust and innovative vaccine effectiveness	Public private partnership	Effectiveness studies of influenza vaccines	https://www.drive-eu.org/	8,999,812.50
I-MOVE+	Integrated Monitoring of Vaccines in Europe: a platform to measure and compare effectiveness and impact of influenza and pneumococcal vaccines and vaccination strategies in the elderly	Translational collaborative research	Comparative effectiveness, impact of vaccines	http://www.i-moveplus.eu/	7,482,729
FluPRINT	Tracing the influenza vaccine imprint on immune system to identify cellular signature of protection	Training and mobility	Cellular immune response to influenza vaccine in children	https://cordis.europa.eu/project/rcn/215465_en.html	269,858
UNIFLUVAC	A novel universal influenza vaccine targeting epitopes of limited variability	Frontier research	Epitopes of limited variability in novel influenza vaccine	https://cordis.europa.eu/project/rcn/218373_en.html	149,297

*MVA, modified vaccinia virus Ankara; VLP, virus-like particle.

**Figure:**
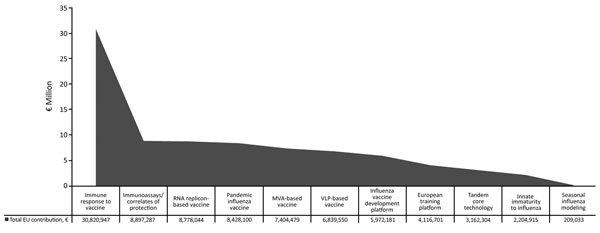
Funding from the seventh Framework Programme for Research and Technological Development of the European Union to influenza vaccine development. EU, European Union; MVA, modified vaccinia virus Ankara; VLP, virus-like particle.

Projects in this area have sponsored basic investigator-driven research to study immunity against influenza virus (e.g., INIMIN, IMMUNExplore), together with projects disentangling the immune response to influenza vaccines (Table 1). Among these, FERFLU EXPRESS investigators conducted de novo sequencing of the transcriptome in ferrets, unveiling >1,000 genes that were involved in the innate and adaptive immune response against influenza. Researchers also discovered promising genetic biomarkers that could lead to novel diagnostics to detect highly pathogenic influenza strains. European immunologists working in TCDIFLU analyzed the immune response of mice vaccinated with H1N1 with or without adjuvant, which resulted in different counts of CD8 T cells, the presence or absence of memory T cells, and different gene expression profiles. This preclinical work may clarify the mode of action of influenza vaccines and their immunogenicity, and could be used in the selection and alteration of vaccine candidates to evoke the desired immune response.

The high-impact FP7 project ADITEC was launched in 2011 to create new technologies for the development of the next generation of human vaccines (*26*). ADITEC has made considerable advances in new immunization technologies, adjuvants, vectors and delivery systems, formulations, and vaccination methods targeting different age groups. The project has particularly focused on influenza, conducting the earliest reported systems biology study of an adjuvanted and nonadjuvanted influenza vaccine in infants and a subsequent clinical trial. Results from this research have provided insights into the innate and adaptive responses to influenza vaccination in early childhood and revealed potential correlates of the antibody response.

In the field of pandemic influenza, the investigators in the frontier research project FLUPAN (funded by an advanced grant of the European Research Council) have developed an HA vaccine repository that is continuously updated with representative virus strains that correspond to circulating influenza viruses of animal origin with pandemic potential. This repository enabled the development of a vector-based vaccine candidate using a modified vaccinia virus Ankara that elicits an immune response against the newly emerging A(H5N8) virus. An efficacious and protective vaccine can be quickly developed in response to a future influenza pandemic menace (*27**,**28*). The collaborative project NASPANVAC focused on developing an intranasal pandemic influenza vaccine that stimulates both local and systemic immune responses. Several studies in animal models have led to the development of a chitosan-adjuvanted vaccine formulation and have demonstrated an association of severity of A(H5N1) disease and protective outcomes with the route of immunization (*29*).

Hemagglutination inhibition titers have been the gold standard for correlates of protection and influenza vaccine efficacy for regulatory approval. However, the correlate of protection for next-generation influenza vaccines may not rely on hemagglutination-inhibiting antibodies (*21*), and broader humoral and cellular protective immune responses warrant further studies (*30**,**31*). To this end, the FP7-funded project IMECS focused on defining the characteristics of protection against avian and pandemic influenza (*32*). The researchers studied the cellular, humoral, and innate immune responses to pandemic influenza A(H1N1) and avian influenza A(H5N1) in different population subgroups such as children and the elderly. Findings from IMECS revealed that seasonal influenza vaccination could induce cross-reactive antibodies against H1N1 pandemic virus and potential H5N1 pandemic strains, particularly in young adults, whereas the elderly showed impaired immune responses. The consortium also assayed several peptide vaccine candidates with encouraging results.

The development of broadly protective influenza vaccines requires parallel standardization of validated immunoassays to measure the immune correlates of protection (*30*,*33*,*34*). The project FLUCOP, sponsored by the Innovative Medicines Initiative, the largest public–private partnership in the world in life sciences (total budget €5.3 billion for 2008–2024) (*35*), will produce a standardized toolkit to evaluate the immune response triggered by new influenza vaccines. The ultimate goal is to ensure that the results of clinical studies of influenza vaccines are comparable across settings, which has important ramifications for regulatory pathways and the development quality of influenza vaccines. Recently, FLUCOP has demonstrated that LAIV has the ability to confer cross-protective immunity to drifted and potentially heterovariant strains, which might lay the foundation for a truly universal influenza vaccine (*36*).

A universal vaccine will likely take many years to achieve, and training the next generation of influenza researchers who will keep working toward this goal is essential. The Marie Curie action UNIVACFLU is establishing a training platform in Europe for young researchers with a novel multidisciplinary approach to develop mucosal influenza vaccines (*37*). Using this approach, recent studies of the delivery of nanoparticle protein formulations to the nasal mucosa successfully demonstrated an immune response without crossing the airway epithelial barrier, pointing to nanoparticles as a potentially effective means to administer mucosal vaccination.

In 2013, in one of the most direct efforts to improve influenza vaccines, the European Commission funded work to develop influenza vaccines that would confer longer-lasting and broader protection than current seasonal influenza vaccines. Five consortia (EDUFLUVAC, FLUNIVAC, FLUTCORE, UNISEC, and UNIVAX) with total funding of €25.5 million began work, with the ultimate goal to develop influenza vaccines that protect from both seasonal and pandemic influenza.

EDUFLUVAC has worked on the principle of strain-specific epitope dilution performed for influenza antigens using viruslike particles (*38*). The consortium is currently conducting a preclinical proof of concept for a vaccine candidate on ferret and nonhuman primate models. The project has also made efforts to standardize immunoassays (*33*), animal models, and common infrastructures for broadly protective influenza vaccine development.

The UNIVAX consortium is engaged in developing multimeric and synthetic nanoparticle-based RepRNA (replicons derived from a noncytopathic porcine pestivirus) influenza vaccine. This candidate vaccine elicits both humoral and cellular immune responses in animal models. Additional developments are nanoparticle formulations for delivery to dendritic cells (*39*) and the evaluation of several adjuvants that induced humoral and cellular cell responses in preclinical and clinical studies. A clinical trial has been conducted with intranasally administered live attenuated influenza vaccine in children and adults to define immunological correlates (*36**,**40*).

Researchers in FLUTCORE have developed a tandem core vaccine platform for a universal influenza A vaccine. This technology forms viruslike particles (derived from hepatitis B virus core protein) carrying several conserved universal influenza antigens. FLUTCORE has completed a proof of concept showing immunity to conserved influenza A virus antigens in mouse models (*41*).

FLUNIVAC has designed a vaccine candidate based on a modified vaccinia virus Ankara. The vector has been demonstrated to be safe and to induce both humoral and cellular immune responses, proving suitable for the production of multivalent vaccines, high-production capacity, and long-term storage (*42*). Ideally, the vaccine is expected to confer protection against seasonal and pandemic influenza A, and it can be rapidly produced in a developed avian cell line, removing the need for passage in chicken embryo fibroblasts and thus avoiding potential adaptive mutations that may limit vaccine effectiveness (*43*).

The UNISEC network has developed and conducted experiments of new vaccine candidates including peptide-based vaccines, DNA-based vaccines, vector-based vaccines, and vaccines combined with different adjuvants in various animal models. Several vaccine candidates have been formulated as dry powder, enabling prolonged storage, cold-chain independence, and new administration routes. The partner BiondVax has created M-001, a major vaccine candidate consisting of 9 conserved epitopes and designed to prevent seasonal and pandemic influenza. M-001 has been tested in a Phase 2b trial in collaboration with UNISEC (*44*). The promising results have led to a loan of €20 million granted to BiondVax by the European Investment Bank through the Horizon 2020 InnovFin Infectious Diseases (*45*). This new loan facility offers financing for high-risk infectious disease projects, in a field where nondilutive funding can be very challenging for SMEs to obtain. BiondVax has successfully completed the recruitment in a first season of a pivotal clinical efficacy phase 3 trial of the M-001 universal flu vaccine candidate, and has also set up a midsized commercial manufacturing plant.

Vaccine hesitancy and misconceptions add to technical challenges. Renewed efforts on social sciences research to tackle vaccine resistance and misperceptions are crucial to the success of new influenza vaccines. The FP7-funded ECOM@EU project has investigated determinants affecting vaccination uptake such as community perception and official communication about risk, developing an evidence base for policymakers that can be adapted for each country in Europe. Specifically, ECOM@EU studied risk perception to pandemic influenza and found that it varies geographically and over time, and so responses need to be adjusted accordingly (*46*).

The consortium TELL ME focused on outbreak communication strategies to maximize vaccine uptake and to assist health professionals and agencies to engage with vaccine-resistant groups (*47*). This project uses the 2009 pandemic of influenza A(H1N1) as their reference scenario.

Successful development of new vaccines also relies on gathering data each year on the effectiveness of existing vaccines. Sound evidence of adequate protection of current influenza vaccines is missing, particularly in persons at risk and persons >65 years of age (*9*). The Innovative Medicines Initiative project DRIVE, within the Horizon 2020 Health Programme, will develop a platform to evaluate this in Europe each year, and this effort is following on from the successful work by the I-MOVE+ project, which has built a large network across the continent to measure comparative effectiveness and impact of influenza vaccines to inform public health policymakers (*48*). I-MOVE+ builds on the success of its predecessor I-MOVE that has repeatedly reported on the suboptimal performance of inactivated influenza vaccine against influenza A(H3N2) finding, for instance, a vaccine effectiveness of 23.4% in the >65 age group for the 2016–17 season (*49*).

## Addressing the Challenges to Seize Opportunities for Public Health

The EU's political commitment in support of immunization is laid out in several conclusions and recommendations of the European Council (*50**–**52*). As of December 2018, the Council of the EU has approved a recommendation on vaccines, driven by a renewed impetus to engage EU member states in the fight against vaccine-preventable diseases.

To feed into this process, in June 2017 the European Commission organized a workshop on next-generation influenza vaccines. The aim of this meeting was to take stock of progress made in the field, identify technical hurdles in the development of these vaccines, and consult a wide range of experts on unsolved regulatory issues and public health challenges. Participants included researchers, academics, clinicians, EU project coordinators and partners, delegates from the pharmaceutical industry and biotechnology companies, public health authorities, policy and decision makers, and representatives from international and philanthropic organizations. Technical, regulatory, and public health challenges for new influenza vaccines were identified in discussions (Table 2). Major conclusions from the workshop included the need for early regulatory advice to ensure adaptation of novel influenza vaccines to individual responses and processes, and prompt interaction with epidemiologists and public health specialists to meet public health needs. Influenza was strongly underlined as a truly global health threat, and participants discussed how issues like regulatory frameworks, global funding, and technology transfer for research and development play a role in the global burden of influenza. Participants discussed EU vaccine policy activities within the Joint Action on Vaccination (*53*) such as immunization information systems, vaccine forecasting and implementation strategies.

**Table 2:** Challenges in the roadmap for the next-generation influenza vaccines

Challenge types
Technical
Eliciting adequate cross-protective humoral and cell-mediated immune response
Selection and delivery of immunogens
Alternatives to egg-based vaccine production
Immunosenescence, “inflammaging” (adjuvants, high-dose vaccine, alternative routes of delivery)
Optimal animal models
Vaccine profiling for different target groups, priming, booster, etc.
New approaches for elucidation of mechanistic aspects of vaccine safety and efficacy (i.e., systems vaccinology)
Regulatory
Design of optimal placebo-controlled clinical trials (when ethical) and with active comparator
Identification of immune correlates of protection
Harmonization and standardization of immunoassays to measure correlates of protection
Human challenge studies
Prospective longitudinal cohort studies for evaluation of influenza immunity and effectiveness of influenza immunization
Planning for postmarketing surveillance
Development cost
Public health
Selection of meaningful endpoints: preventing severe or overt disease vs. blocking infection or transmission
Defining target groups and vaccination schemes
Logistics of vaccine implementation and delivery
Vaccine access
Vaccine supplies and stockpiling
Influenza vaccination for resource-poor settings
Influenza disease and vaccine misperceptions;
Vaccine hesitancy
Funding mechanisms for vaccine development
Cost-effectiveness of universal influenza vaccines

Having heard the issues raised in these discussions, at this stage of next-generation influenza vaccine development, the European Commission is working to launch new funding, such as the joint call with the Department of Biotechnology of India devoting €30 million to develop a next-generation influenza vaccine (*54*). An expected impact of this vaccine is to reduce the burden of influenza worldwide and contribute to Sustainable Development Goal 3 (*55*), targeting specific populations in India and Europe. In doing so, applicants may address WHO Preferred Product Characteristics for next-generation influenza vaccines.

The development path to new influenza vaccines is paved with challenges but offers numerous opportunities for the betterment of the world’s health (Table 3). An improved vaccine is one important facet, but many other issues also require focus from international partners. For instance, faster and continuous vaccine production will improve supply and stockpiling; needle-free delivery systems will enable better uptake of the vaccine, and better formulations (e.g., thermostable) and lower implementation costs derived from longer-lasting immune response will help expanding influenza vaccine coverage, particularly in resource-limited settings. In addition, indirect and generalized herd effects of the vaccine will grant protection at population levels beyond individual efficacy (*56**,**57*), and improved advocacy and education of healthcare professionals and the public will combat vaccine hesitancy and misperceptions (*58*).

**Table 3:** Opportunities in the development of the next-generation influenza vaccines

Opportunity
Faster manufacturing process
Continuous production: improving supply and stockpiling
Technology transfer to meet the implementation demand
Needle-free delivery systems
Lower implementation costs (doses, regimen, delivery)
Enhanced effectiveness in the elderly
Better uptake of the vaccine
Expanding influenza vaccine coverage in resource-poor settings
Indirect and generalized effects of the vaccine
Better arguments for advocacy and education of healthcare professionals and general public
Influenza pandemic preparedness planning
Age-personalized approach to vaccine development
Facilitating pandemic preparedness and response
One Health approach

The road to a truly universal influenza vaccine might be long, and is in fact part of a stepwise process made up of several intermediate improvements. As a global research funder, the EU is making every effort to foster next-generation influenza vaccine development by encouraging transnational collaboration, new technologies, and regulatory frameworks with a broad public health perspective. It is important that we politically commit to protect European citizens’ health and to continue to take these steps until influenza ceases to be a scourge on the world's health.
